# Effectiveness of Near-Peer-Taught Case Reviews on Students' Confidence in National Board of Medical Examiners (NBME) Exams

**DOI:** 10.7759/cureus.43661

**Published:** 2023-08-17

**Authors:** Ashley Mason, Charles Jang, Katsiaryna Khatskevich, Zeegan George, Caleb Streitmatter, Britton McGlawn-McGrane, Jessica Dominguez Rieg

**Affiliations:** 1 Medical School, University of South Florida Morsani College of Medicine, Tampa, USA; 2 Department of Emergency Medicine, Wright State University, Dayton, USA; 3 Department of Pathology and Laboratory Medicine, Medical University of South Carolina, Charleston, USA; 4 Department of Internal Medicine, Medical University of South Carolina, Charleston, USA; 5 Department of Molecular Pharmacology and Physiology, University of South Florida Morsani College of Medicine, Tampa, USA

**Keywords:** nbme examinations, problem-based learning, medical curriculum, case-based learning, medical education, near-peer teaching

## Abstract

Introduction

A key element to a first-year medical student's (MS1) education is guidance and practice in applying anatomy, pathophysiology, diagnosis, and treatment concepts to clinical vignettes. One potential solution to providing effective clinical reasoning training is the involvement of second-year medical students (MS2s) in small group sessions as teachers to provide more personalized instruction via case-based learning sessions. Near-peer teaching has been shown to benefit students' confidence in learning and improve test scores. Similarly, case-based learning is heavily associated with an improved understanding of complex topics. As such, this study assessed the efficacy of near-peer teaching with concomitant case-based style presentations on improving the comfort of MS1s with their understanding of the curriculum content and their comfort with applying their knowledge to clinical scenarios.

Methods

This randomized controlled crossover trial included several small-group study sessions, each consisting of five MS1s and led by an MS2 who reviewed selected clinical cases in a standardized slide decks. The control arm was provided the same slide decks but did not participate in the MS2-led sessions. During the first course, students were assigned to either the control or intervention group and then crossed over to the opposite group (control to intervention and vice versa) in every subsequent course. Comfort with the curriculum material was then assessed through pre and post surveys, with the post surveys administered after the MS1s took their final NBME examination for that course.

Results

The study was cut short due to COVID-19 precautions limiting in-person sessions. Nevertheless, the post survey demonstrated an increased understanding of pathological concepts for the intervention group compared to the control group.

Conclusions

Future work on near-peer group study sessions should enroll a larger sample size with measures to improve the response rate to better test whether near-peer-led case reviews had a significant effect on students' understanding of anatomical concepts and confidence during NBME examinations.

## Introduction

Medical schools aim to teach students to thoroughly assess and treat their patients' presenting illnesses. To accomplish this goal, schools have implemented various examinations to assess students' strengths and weaknesses and to improve students' clinical problem-solving over the first year of medical school. One common form of assessment is the National Board of Medical Examiners (NBME) examinations. The NBME Basic Science Subject Exams are subject-based exams that utilize USMLE (United States Medical Licensing Exam)-style questions to assess students' knowledge of core anatomical, pathological, and physiological concepts. These multiple-choice questions utilize a clinical vignette; patient age, gender, pertinent medical history, and chief complaint are provided with the aim of assessing students' ability to synthesize multiple data points together to arrive at the correct diagnosis. Improved scores on NBME examinations have been linked with improved USMLE Step 1 examination scores. In contrast, poor scores helped identify students struggling academically with the first-year curriculum [1].

One growing technique for teaching medical students how to integrate NBME-style problem-solving is the case-based approach; real-world clinical cases are used to demonstrate major medical concepts and teach clinical reasoning skills [2]. Case-based learning is increasingly common in medical curricula and healthcare settings, in part due to the ease of delivery through various formats such as in-person, online, or paper formats [2]. In regard to anatomy education, students have reported increased satisfaction with anatomy lessons when case-based images are integrated into lectures [3,4]. In addition, case-based learning has shown benefits in pathology learning, improved motivation to learn about pathological subjects, and examination scores [5]. Replacement of traditional didactics with case-based small-group sessions has been shown to significantly improve NBME examination scores [6]. Overall, case-based learning provides a collaborative environment that can improve examination scores as well as increase student confidence in discussing and problem-solving complex medical illnesses with peers [7].

Alongside case-based approaches, near-peer education, or education of students by fellow students, serves as a potent tool for improving students' outcomes. Near-peer tutors have been associated with increased student satisfaction by first-year medical students and an increased understanding of the subject matter by both the upperclassmen tutors and knowledge recipients [8-11]. In one study by Diebolt et al., near-peer teaching of surgical anatomy helped increase third-year students' sense of self-efficacy and anatomical knowledge compared to traditional medical education practices such as didactics [12]. In addition, first- and second-year medical students have been shown to have improved scores in anatomy examinations when they were taught by near-peers, specifically upperclassmen, through anatomical review sessions in cadaver laboratories [13,14]. Similarly, weekly near-peer teaching sessions in the second year of medical school have been shown to correlate with higher USMLE Step 1 examination scores [15]. 

Due to the benefits discussed, this study aimed to explore how the near-peer teaching model, in the form of small group case-based teaching sessions taught by upperclassmen, could influence students' confidence and understanding of medical education concepts. Studies have shown that increased self-efficacy, or in other words increased self-rated confidence, is correlated with increased cognitive engagement with subjects [16,17], and therefore improved confidence may have direct benefits to students’ engagement with the curriculum. Additionally, this study evaluated whether such sessions improved students' confidence in solving USMLE clinical vignette-style questions and passing NBME-style examinations, as well as improved student understanding of anatomical, physiological, and pathological course contents. 

This article was previously presented as an abstract and poster at the Florida Medical Association's 2022 David A. Paulus, MD Poster Symposium on August 6th, 2022.

## Materials and methods

Study design

This open-label crossover trial was advertised to all first-year medical students (MS1s) at the University of South Florida Morsani College of Medicine through an announcement at the students' first-year orientation as well as through social media at the beginning of the 2019-2020 academic year. Students were included if they were part of the first-year class of medical students (MS1s) and excluded if they had previous experience in medical school, such as repeating a year or transferring from another medical school. Forty students registered and were randomly divided into intervention and control groups for Basic Medical Sciences 1: Musculoskeletal System (Course 1). The intervention and control groups were swapped for Basic Medical Sciences 2: Neurological System (Course 2) and again for Basic Medical Sciences 3: Cardiovascular and Pulmonary Systems (Course 3). Due to COVID-19 affecting in-person educational activities in March 2020, this study was terminated prior to the MS1s' 4th and final course of the first year (Basic Medical Sciences 4: GI, Endocrine, Renal, and Reproductive Systems). 

Case-based instruction implementation

The First Aid Cases for the USMLE Step 1, Third Edition by Le and Yeh [18] and Neuroanatomy Through Clinical Cases, Second Edition by Blumenfeld [19] were used to create a slide decks of clinical vignettes, relevant questions for each case (e.g., diagnosis, pathophysiology, treatment), and answers with explanations. Then, MS2 peers were selected and briefed before small group sessions on the main points of each case, as well as the relevant anatomy, physiology, and pathology, to ensure inter-classroom homogeneity of lessons. After each session, the MS1 participants were provided with the slide decks. These group learning sessions were conducted a week before students' NBME examination at the end of each course. Students within the MS1 control group were provided with the case-based slide decks at the same time as the intervention group but did not receive any instruction from the second-year near-peer teachers. 

Evaluation of sessions

Pre and post surveys were administered anonymously through the Qualtrics XM software for all three courses. Pre surveys were administered to both groups before each session, while post surveys were administered after the NBME examinations. Students were asked to rate their comfort levels with anatomy, physiology, and pathology, as well as their confidence in answering USMLE-style vignette questions and taking the NBME examination itself. Answers were on a quantitative agreement scale, with one being the lowest option and five being the highest. Internal validity of the survey was determined through calculation of Cronbach’s alpha, with alpha ≥ 0.70 considered acceptable.

Statistical analysis

Average bipolar Likert scale responses and standard deviations for each course pre survey and post survey for both the intervention and control group were calculated as well as the average Likert response and standard deviation across all three courses. These were obtained by converting the scale that ranged from 1 to 10, with 1 being strongly disagree to 10 being strongly agree to a 5-point scale where the numbers 1 and 2 represented strongly disagree (1 on the new scale), 3 and 4 represented slightly disagree (2), 5 and 6 represented neutral (3), 7 and 8 represented slightly agree (4), and 9 and 10 represented strongly agree (5). Changes in average Likert responses between pre and post surveys for each course, as well cumulatively, for both the intervention and control groups, were calculated and reported to three decimal places. The results of the post surveys for the intervention and control groups were then compared. A chi-square analysis was performed to confirm that the intervention and control group data did not come from significantly different distributions. Levene's test for Homogeneity of Variances was calculated to determine that the data met the assumptions of the Unpaired T-test. Unpaired T-Tests were used to compare the average post survey responses for the intervention and control groups across all three courses. All results were reported with an alpha of 0.05. Statistical analysis was performed using Microsoft® Excel for Mac Version 16.68 and SPSS 28.

## Results

**Figure 1:**
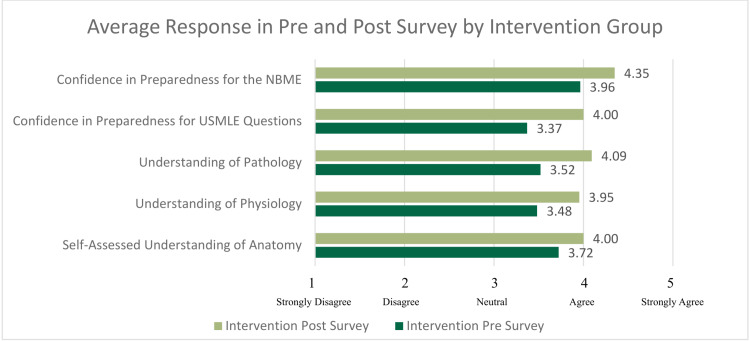
Average responses per question for all three courses for students in the intervention group Students were asked to rate their confidence and understanding on a scale of 1 (strongly disagree) to 5 (strongly agree). Strongly agree corresponds to higher self-rated confidence. NBME: National Board of Medical Examiners; USMLE: United States Medical Licensing Exam

**Figure 2:**
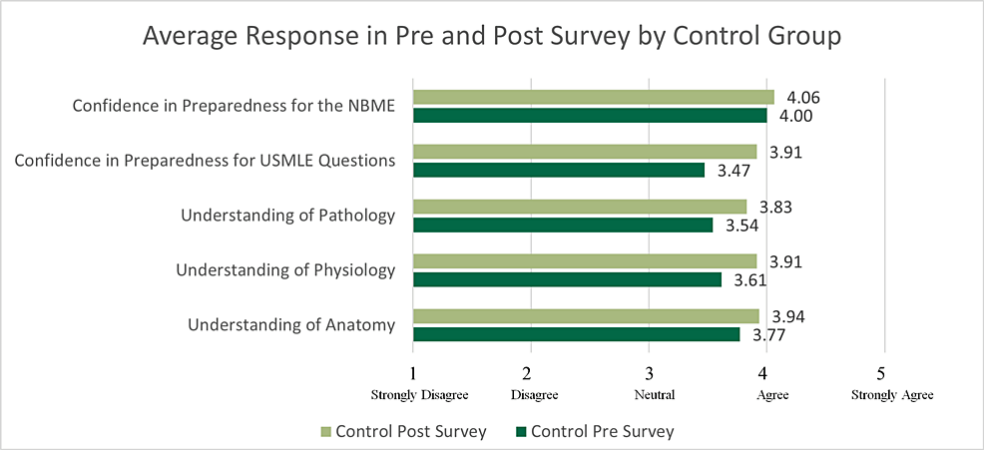
Students in the control group's average responses per question for all three courses Students were asked to rate their confidence and understanding on a scale of 1 to 5; 5 being strongly agree and corresponding to higher self-rated confidence. NBME: National Board of Medical Examiners; USMLE: United States Medical Licensing Exam

Overall, 40 MS1s participated in the study, producing 110 pre survey responses and 99 post survey responses over the three courses for which data were collected. Within the control group, 56 pre survey and 47 post survey responses were collected. Within the intervention group, 54 pre survey responses and 52 post survey responses were collected. The number of participants responding to both the pre and post surveys decreased each course. The average response per question of the control and intervention groups are shown in Figures 1, 2, and Table 1. To assess internal validity, Cronbach’s Alpha was calculated for the pre and post survey results in the control and intervention group and was 0.88. See supplemental Figure 3 for an additional diagram showing changes in responsiveness to the survey by course.

**Table 1:** Average responses to the Likert scale questions evaluating students' self-assessed confidence by course for the intervention and control groups Course 4 was not assessed due to COVID-19 prematurely terminating this study. Responses were rated on a 5-point Likert scale of 1 (strongly disagree), 2 (disagree), 3 (neutral), 4 (agree), and 5 (strongly agree). NBME: National Board of Medical Examiners; USMLE: United States Medical Licensing Exam

	Confidence in Preparedness for USMLE-Style Questions		Confidence in Preparedness for the NBME exam		Understanding of Anatomy		Understanding of Physiology		Understanding of Pathology	
	Control	Intervention	Control	Intervention	Control	Intervention	Control	Intervention	Control	Intervention
Average C1 Pre Survey (n control=20, n intervention=20)	3.60±0.68	3.35±0.88	4.00±0.86	3.75±1.02	3.95±0.69	3.55±0.76	3.95±0.69	3.45±0.69	3.60±0.75	3.35±0.75
Average C1 Post Survey (n intervention=20, n control=17)	4.00±1.00	3.70±0.86	4.06±1.09	3.95±1.00	4.24±0.66	3.95±0.60	4.24±0.56	3.75±0.55	3.82±0.73	3.90±0.55
Average C2 Pre Survey (n control=18, n intervention=19)	3.25±0.85	3.40±0.88	3.65±0.81	4.10±0.85	3.25±0.85	3.60±0.82	3.40±0.88	3.60±0.88	3.50±0.76	3.70±0.80
Average C2 Post Survey (n control=19, n intervention=20)	3.94±0.80	4.11±0.74	4.06±0.80	4.63±0.68	3.61±0.78	4.11±0.74	3.61±0.70	4.16±0.69	3.94±0.80	4.16±0.69
Average C3 Pre Survey (n control=17, n intervention=14)	3.59±0.87	3.36±0.74	4.41±0.80	4.07±0.92	4.18±0.73	4.14±0.66	3.47±0.62	3.36±0.74	3.53±0.80	3.50±0.65
Average C3 Post Survey (n control=12, n intervention=13)	3.75±1.22	4.08±0.76	4.08±1.08	4.38±0.77	4.00±0.95	3.92±0.86	3.92±1.24	3.92±0.64	3.67±0.89	4.15±0.55

**Figure 3:**
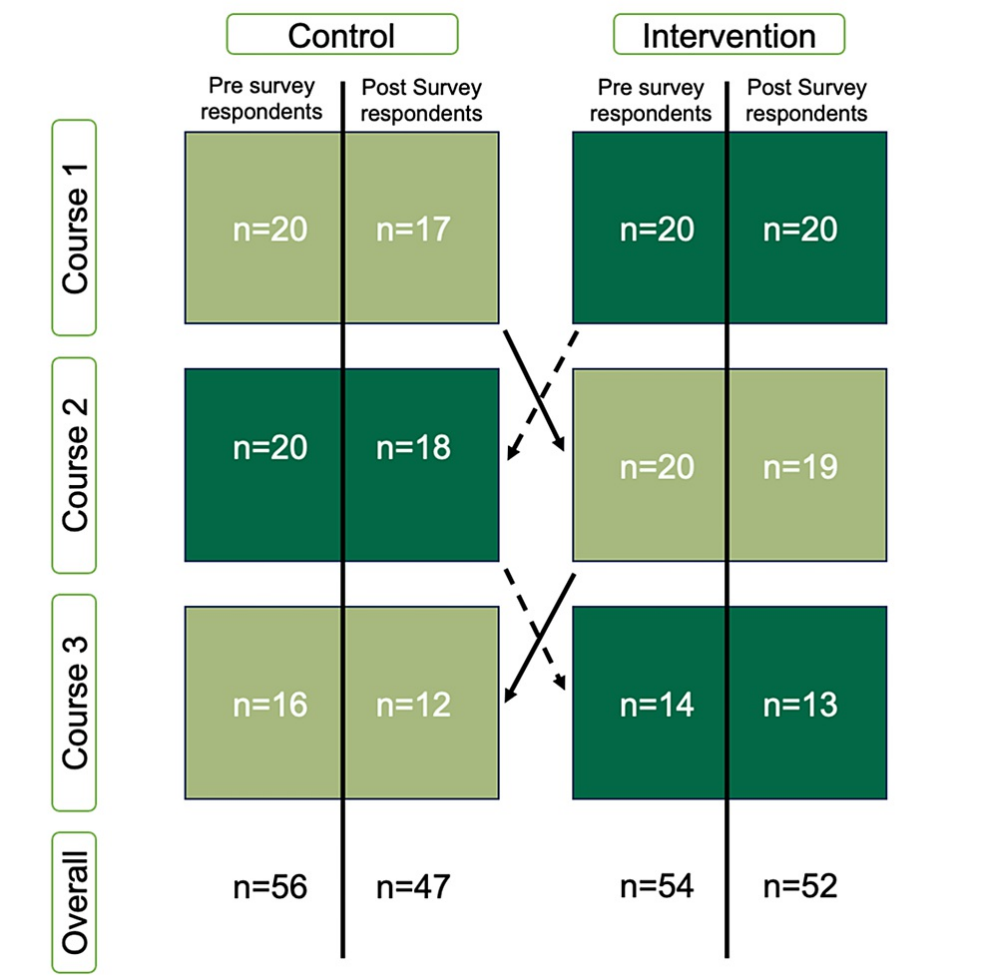
Changes in responses over time As demonstrated, during crossover, not all respondents responded to each pre and post survey administration. Light green represents the group that initially served as the control group during Course 1, while dark green represents the group that served as the intervention group during Course 1. n represents the number of survey respondents for each survey iteration.

The distributions of responses were not found to be significantly different between the intervention and control groups (χ2=2.213, p=0.331), permitting the unpaired t-test to be utilized. Students in the intervention group reported a statistically significant increased self-assessed understanding of pathological concepts (t(97)=-1.598, p=0.0114) compared to the control group's post survey responses. However, students in the intervention group did not differ significantly from the control group on the post survey in terms of self-assessed understanding of anatomical concepts (t(97)=-0.414, p=0.679) or physiological concepts (t(97)=-0.182, p=0.856). In addition, students receiving the intervention did not have any statistically significant difference in the students' confidence to apply their knowledge in answering USMLE-style questions (t(97)=-0.153, p=0.878) or confidence in their ability to pass their NBME examinations (t(97)=-1.319, p=0.190) compared to the control group.

Changes in average reported responses between pre and post surveys

When comparing the average change in Likert responses from pre to post survey across all courses by cohort, there was an increased self-rated understanding of anatomical concepts (+0.081) and increased self-rated understanding of pathological concepts (+0.043) among the intervention group. There was also a positive change in confidence to pass the NBME (+0.092) in the intervention group compared to the control. However, there was a negative trend in their self-rated understanding of physiological concepts in the intervention group compared to the control group (-0.088). Similarly, there was a negative average change in confidence to answer USMLE-style questions with clinical vignettes in the intervention group compared to the control (-0.109).

## Discussion

This study demonstrated that case-based teaching by near-peers was correlated with an increase in students' self-assessed understanding of pathological concepts within the intervention group compared to the control group, matching existing literature showing that case-based learning may improve students' understanding of the curriculum regardless of the delivery method [2, 8] and specifically within pathology lessons [5]. Our findings are also consistent with literature showing that near-peer approaches to education can improve understanding of subjects [8-11]. The participants in this study reported statistically significant improvements in confidence, suggesting the combined intervention of near-peer tutoring and case-based sessions could serve as a tool to improve MS1 students' confidence in pathological concepts. This is consistent with prior literature that has demonstrated that case-based approaches can improve student confidence in various metrics [7], and near-peer-based education has similarly been correlated with increased self-efficacy [12].

Interestingly, there was lower self-rated confidence in understanding physiological concepts and in answering USMLE-style questions when comparing the intervention versus control groups. This contradicts prior literature that has shown near-peer taught lessons can increase understanding of various concepts such as anatomy [3,4] and pathology [5] regardless of the format of instruction [2]. There are multiple possible reasons for this, all necessitating further research. Students may have rated their confidence lower due to having participated in a group session and feeling less knowledgeable than their peers. If the decrease in confidence were due to the students' perception of a gap in their fund of knowledge in comparison to their peers and the near-peers, this may not necessarily have correlated with performance on the exam. Another explanation could be that the group sessions taught by near-peers may include higher-level information (such as information covered in the second-year curriculum) volunteered by the near-peer teachers. This could have led to a negative impact on students’ confidence in understanding concepts and answering questions. Future studies can assess whether the lower confidence in the intervention group may be associated with students’ better ability to identify weak points, or alternatively how to counteract this lower confidence. In addition, future studies can assess whether these changes in confidence correlate with examination score differences.

This study was limited by a small number of participants, in part due to COVID-19 limiting the length of the study as it did not continue into Course 4 as initially planned. This may have skewed results, as one group of students received interventions in two of the three courses studied and therefore may have had different perceptions in comparison to the group that served as a control for two courses of the study. Another limitation of this study was that the gathered data was limited in scope; data was collected using Likert Scales to assess students' perceived comfort, but quantitative NBME exam scores and course grade data were not collected. Therefore, objective measures of understanding were not ascertained and this study may have been affected by the Dunning-Kruger effect or by overconfidence by participants. 

In addition, due to the anonymous nature of the surveys, participants' responses for the pre and post surveys were unable to be paired; thus, this study was unable to assess the effects of the intervention at the granularity of the individual participant. Future work on this topic should enroll a larger sample size and be designed to maximize the response rate to avoid the aforementioned pitfalls experienced in this study. In addition to the outcome of student comfort, test grades would also be beneficial to measure to provide an objective performance metric. Studies have shown that case-based learning can be associated with improved pathology examination scores [5] and improved NBME examination scores [6]. In addition, near-peer education has been correlated with increased scores in anatomy examinations [13,14] and improved USMLE examination scores as well [15]. Due to the benefits of case-based learning and near-peer-based sessions for confidence and examination scores, further exploration of their direct benefits on objective measures such as examination scores is integral to helping improve student success within the first-year curriculum.

## Conclusions

Near-peer teaching through case-based review may be an effective method for helping students gain confidence in answering NBME-style questions and understanding anatomical concepts. However, further research with more participants is needed to better elucidate the specific benefits of similar sessions within the medical curriculum. This study demonstrated that implementing a case-based near-peer teaching method for NBME-style examinations is a viable curriculum intervention for MS1s. In addition, it showed that similar study sessions may be a beneficial core or supplemental addition to the medical school curriculum for MS1s, and can effectively improve their understanding of medical concepts, specifically physiological concepts in the intervention group in comparison to the control group. It can also boost their confidence in basic medical concepts, which is integral as studies have shown that increased self-efficacy can in turn result in increased engagement within the medical field.
